# The complete mitochondrial genome of a cold seep gastropod *Phymorhynchus buccinoides* (Neogastropoda: Conoidea: Raphitomidae)

**DOI:** 10.1371/journal.pone.0242541

**Published:** 2020-11-30

**Authors:** Lvpei Du, Shanya Cai, Jun Liu, Ruoyu Liu, Haibin Zhang

**Affiliations:** 1 Institute of Deep-sea Science and Engineering, Chinese Academy of Sciences, Sanya, China; 2 University of Chinese Academy of Sciences, Beijing, China; Universiti Malaysia Sabah, MALAYSIA

## Abstract

*Phymorhynchus* is a genus of deep-sea snails that are most distributed in hydrothermal vent or cold seep environments. In this study, we presented the complete mitochondrial genome of *P*. *buccinoides*, a cold seep snail from the South China Sea. It is the first mitochondrial genome of a cold seep member of the superfamily Conoidea. The mitochondrial genome is 15,764 bp in length, and contains 13 protein-coding genes (PCGs), 2 rRNA genes, and 22 tRNA genes. These genes are encoded on the positive strand, except for 8 tRNA genes that are encoded on the negative strand. The start codon ATG and 3 types of stop codons, TAA, TAG and the truncated termination codon T, are used in the 13 PCGs. All 13 PCGs in the 26 species of Conoidea share the same gene order, while several tRNA genes have been translocated. Phylogenetic analysis revealed that *P*. *buccinoides* clustered with *Typhlosyrinx* sp., *Eubela* sp., and *Phymorhynchus* sp., forming the Raphitomidae clade, with high support values. Positive selection analysis showed that a residue located in *atp6* (18 S) was identified as the positively selected site with high posterior probabilities, suggesting potential adaption to the cold seep environment. Overall, our data will provide a useful resource on the evolutionary adaptation of cold seep snails for future studies.

## Introduction

Conoidea are venomous marine gastropods in Neogastropoda [1, 2], which are found in all oceans, from the tropics to the poles, and from shallow waters to abyssal depths [3]. The superfamily includes 15 families (Borsoniidae, Bouchetispiridae, Clathurellidae, Clavatulidae, Cochlespiridae, Conidae, Conorbidae, Driliidae, Fusiturridae, Mangeliidae, Marshallenidae, Pseudomelatomidae, Raphitomidae, Terebridae, Turridae) [4, 5], and has more than 300 accepted genera and about 5,000 species in total [3, 6, 7]. Raphitomidae, elevated to a full family by Bouchet et al. in 2011 [4], is the largest and most diverse family of Conoidea [8]. Phylogenetic relationships and evolution of Conoidea are very challenging because of its high diversity [9]. In this context, the complete mitochondrial genome analysis can bring new information to the phylogenetic analysis of Conoidea.

For most molluscs, the mitochondrial genome is a closed circular DNA molecule ranging from 15 Kbp to 20 Kbp in length [10], which generally contains 37 genes: 13 protein-coding genes (PCGs) (*cox1-3*, *nad1-6*, *nad4L*, *atp6*, *atp8* and *cob*), 2 encoding ribosomal RNA genes (*rrnS* and *rrnL*), and 22 encoding transfer RNA genes (tRNAs) [10, 11]. In recent years, mitochondrial genome sequences have been widely used in phylogenetic reconstruction and species identification for many marine animal groups [11, 12]. In addition, as energetic centers of cells, all the 13 mitochondrial PCGs are involved in the oxidative phosphorylation, and mutations in these genes can directly influence metabolic performance [10, 13]. Increasing evidence has shown that mitochondrial PCGs are subject to positive selection in response to extreme environmental stress. For example, selective signatures have been detected for mitochondrial PCGs in marine animals inhabiting extreme environments: the *nad5* and *nad2* in Pacific salmon [14], the *nad*2 and *nad*4 in deep-sea sea cucumber [15], the *atp8* and *nad5* in deep-sea Starfish [13], and the *cox1*, *cox3*, *cob*, *nad2*, *nad4* and *nad5* in cold seeps clams [16].

Cold seeps is one of the extreme deep-sea environments, where fluid migrates upward from deep stratum to the seafloor under pressure that result from plate subduction or gravity compression [17–19]. It mostly occurs in geologically active and passive continental margins and trenches [19]. This environment is characterized by darkness, high hydrostatic pressure, variable temperatures and high levels of toxins [16, 20]. Despite the harsh conditions, dense communities of fauna have been observed in the cold seep ecosystems, which are supported by chemosynthetic symbionts [21]. Cold seep communities have a high level of endemism with common specific lineages at levels of family, genus and species [22]. Evidence of adaptations has been found in species inhabiting these chemosynthetic environments, such as clams [16], mussels [23], tubeworms [24] and shrimps [25]. Recent studies have also identified potentially adaptive residues in mitochondrial PCGs in cold-seep clams [16].

In the present study, we have reported the mitochondrial genome of *Phymorhynchus buccinoides* Okutani, Fujikura & Sasaki, 1993 in the family Raphitomidae, a gastropod collected from the Haima cold seeps in the South China Sea at depth of 1388 m. This species was first described by Okutani et al. in 1993, based on specimens collected from a cold seep off Hatsushima, Japan (S1 Fig) [26]. Here, we first presented the mitochondrial genome organization, codon usage and gene order information of *P*. *buccinoides*. Phylogenetic relationships between *P*. *buccinoides* and other species from the superfamily Conoidea were examined based on mitochondrial PCGs. Finally, we performed positive selection analyses in order to understand the adaptive evolution of mitochondrial genes in *P*. *buccinoides* to the cold seeps.

## Materials and methods

### Ethics statement

The snails collected in this study required no specific permits. The sampling locations were not privately owned or protected in any way and the collection did not involve endangered or protected species.

### Sample collection and DNA extraction

The specimen (S1 Fig) was collected at depth of 1388 m by Human Occupied Vehicle “ShenHaiYongShi” during an expedition in Haima cold seeps in the South China Sea (16.73°N, 114.46°E) in 2018. The active Haima methane seeps, which have recently been discovered, are located at depths of 1370–1390 m on the northwestern slope of the South China Sea [27]. Methane-derived authigenic carbonates, abundant gas hydrates and chemosynthetic communities are observed in this seep area [27, 28].

The specimen was morphologically identified to *P*. *buccinoides* according to keys of Okutani et al [26]. The sample (voucher no. IDSSE-EEMB-L02) was stored at -80°C in Institute of Deep-sea Science and Engineering, CAS. Total genomic DNA was extracted from preserved foot tissues using the TIANGEN marine animal DNA kit (TIANGEN, China).

### PCR amplification and sequencing

The complete mitochondrial genome of this sample was obtained by PCR amplification. The short fragments of *cox1*, *rrnL* and *nad5* were amplified with primers LCO1490+HC02198 [29], 16sinicioF2+16sfinR [30] and QW58ND5F4+QW58ND5R3 (designed in this study based on the sequences from other closely related species from NCBI), respectively. The new sequences were used to design specific primers, which were combined with the *cox3* (forward), *cox1* (forward and reverse), and *rrnL* (forward and reverse) primers published by Uribe et al. [2] for long PCR amplification. The remaining unknown fragments were amplified by using the new designed specific primers (S1 Table).

The PCR amplifications were carried out using TaKaRa LA Taq® and the thermal cycling was: a denaturing step at 94°C for 5min; 45 cycles of denaturation at 98°C for 10s, annealing temperatures of 40–50°C for 30s and extension at 68°C for 60s per kb; and a final extension step at 68°C for 12min. A total reaction volume of 50 μl included 33.6 μl ddH_2_O, 5 μl 10× LA PCR buffer (Mg^2+^ plus, TaKaRa), 6 μl dNTP mix (2.5 mM each), 2 μl each primer (10 μM), 0.4 μl LA Taq DNA polymerase (5 U/μl, Takara), and 1 μl DNA template (100 ng/μl). For the annealing temperatures see S1 Table. PCR products were confirmed visually on a 1.0% agarose gel (1× TAE) and purified with gel extraction kit (Omega Bio-tek). The purified product was then sequenced on the ABI 3730x1 DNA analyzer (Applied Biosystems Inc.).

### Sequence analysis and gene annotation

Raw sequences were assembled with the program Seqman within the Lasergene software [31]. Then, the mitochondrial genome were preliminarily annotated by the MITOS webserver (http://mitos.bioinf.uni-leipzig.de/index.py) [32]. NCBI BLAST (http://blast.ncbi.nlm.nih.gov/Blast.cgi) and ORF finder (www.ncbi.nlm.nih.gov/projects/gorf/orfig.cgi) were used to identify PCGs. The locations of rRNA genes were determined by alignment with the homologous genes of other species of Neogastropoda. The tRNA genes and their secondary structures were identified by the program tRNAscan-SE 1.21 (http://lowelab.ucsc.edu/tRNAscan-SE/) [33] and ARWEN 1.2.3.c (http://130.235.244.92/ARWEN/) [34]. The mitochondrial genome map was drawn with GenomeVx [35]. The codon usage analysis was estimated with MEGA7.0 [36].The skew values of AT and GC were used to describe the base composition difference between different families of Conoidea, with the following formulaes: AT skew = (A − T) / (A + T) and GC skew = (G − C) / (G + C) [37].

### Phylogenetic analyses

Phylogenetic relationships of families within the superfamily Conoidea were estimated with sequences of mitochondrial genomes. To balance the number of species in each family, 1–4 species (average 3) of one family were used. Finally, a total of 26 conoid species, belonging to 12 families, were analyzed (Table 2). Phylogenetic relationships were constructed by using Bayesian inference (BI) [38] and Maximum Likelihood (ML) [39] methods. *Nassarius festivus* (Nassariidae) (NC_037607) [40] and *Neptunea arthritica* (Buccinidae) (KU246047) [41] were used as outgroups according to previous phylogenetic studies [2, 42]. All mitochondrial genome sequences used in the analyses are shown in Table 2. Multiple alignments of the 13 PCGs were conducted using MEGA v7.0 [36]. Poorly aligned regions and gaps were removed by using Gblocks v0.91b [43] with the default options. Jmodeltest v2.1.7 [44] was used to calculate the best-fit substitution models for each PCGs partition. The best-fit models are shown in S2 Table.

The BI analyses were performed with MrBayes v3.1.2 [45]. Two parallel runs each with four simultaneous MCMC chains were conducted for 5,000,000 generations, sampling every 1000 generations, and the first 25% of generations were removed as burn-in. Convergence was checked in Tracer v1.6 [46] with effective sample size for all the parameters > 200. For ML, we used the software RaxmlGUI v1.3 [47] with the settings “ML + rapid bootstrap”, 1,000 bootstrap replicates and the GTR+I+G model. Visualization of BI tree and ML tree were realized in FigTree v1.4.3 [48].

### Positive selection analysis

Comparing the synonymous/nonsynonymous substitution ratios (ω = *d*_N_/*d*_S_) of genes in different evolutionary lineages provides an important mean for understanding mechanisms and driving forces of gene evolution [49]. ω>1 indicates positively selected where some favorable mutation is being fixed; ω = 1 indicates neutrality; ω<1 indicates purifying selection where most of the non-synonymous mutations were eliminated [50]. We used the “branch models” and “branch-site models” of ‘CodeML’ program in the pamlX package [50, 51] to estimate potential adaptive evolution in the mitochondrial genes of *P*. *buccinoides*. The ML tree was constructed by MEGA v7.0 [36] as the working topology for all CodeML analyses.

The 13 individual and concatenate PCGs dataset were involved in the positive selection analysis, and all the models have corrected the average nucleotide frequency at three codon positions (CodonFreq = 2, icode = 4). In order to compare the selection pressure acting on the mitochondrial genomes of cold seep *P*. *buccinoides* and other 22 species (S3 Table) of Conoidea inhabiting normal seafloor environments, we used the “one-ratio” (M0), “free-ratio” (M1) and “two ratios” models in the “branch models” to estimate the ω (*d*_N_/*d*_S_) ratios [50]. Since positive selection usually acts on a few sites within a short period of evolutionary time [52], the “branch site models” (model A and null model A) were used to detect positive selection affecting individual site of cold seep *P*. *buccinoides*. Bayes Empirical Bayes (BEB) [53] analysis was adopted to calculate the posterior probabilities of the positively selected sites.

## Results and discussion

### Mitochondrial genome content and gene organization

**Fig 1:**
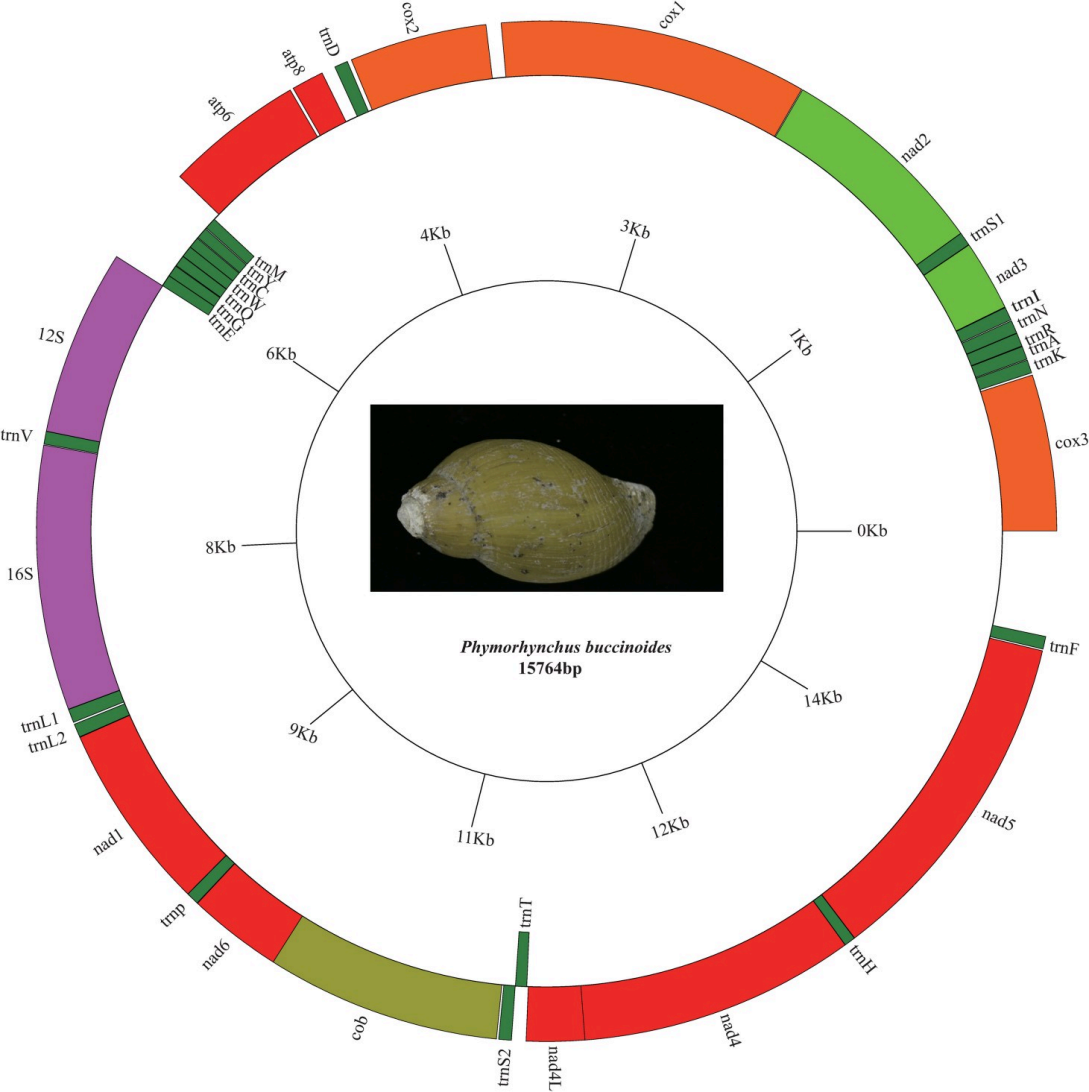
Map of the mitochondrial genome of *P*. *buccinoides*. Genes outside the circle are encoded on the heavy strand (direction 5’-3’) and genes inside the circle are encoded on the light strand (direction 3’-5’). Protein coding genes and rRNAs are shown with standard abbreviation. The tRNAs are designated by a single letter for the corresponding amino acid, with two leucine tRNAs (*trnL1* and *trnL2*) and two serine tRNAs (*trn*S1 and *trnS2*) differentiated by numerals.

The mitochondrial genome of *P*. *buccinoides* is a 15,764 bp circular molecule (Fig 1). The genome comprises 37 genes, including 13 PCGs, 2 rRNA genes, and 22 tRNA genes (*trnL*^CUN^, *trnL*^UUR^, *trnS*^AGN^ and *trnS*^UCN^ is denoted as *trnL1*, *trnL2*, *trnS1* and *trnS2*, respectively). Among them, 29 genes are encoded on the heavy (H) strand, whereas the other 8 tRNA genes are encoded on the light (L) strand (Fig 1 and Table 1). A total of 24 noncoding regions are found (Table 1), and the largest region (519 bp) is between *trnF* and *cox3* (Fig 1 and Table 2) and is identified as the putative control region due to the AT richness (77.64%) (Table 2) and its location [3, 54]. The complete mitochondrion has been deposited in GenBank (GenBank accession ID: MN583349).

**Table 1:** Characteristics of the mitochondrial genome of *P*. *buccinoides*.

Gene	Location		Size		Codon		Intergenic nucleotide(bp)^a^	Strand
	Start	Stop	Nucleotides(bp)	Amino acids	Start	Stop		
*cox3*	1	780	780	260	ATG	TAA	519	+
*trnK*	792	858	67				11	+
*trnA*	863	929	67				4	+
*trnR*	932	999	68				2	+
*trnN*	1001	1066	66				1	+
*trnI*	1072	1138	67				5	+
*nad3*	1141	1494	354	118	ATG	TAG	2	+
*trnS1*	1496	1563	68				1	+
*nad2*	1564	2622	1059	353	ATG	TAA	0	+
*cox1*	2627	4165	1539	513	ATG	TAA	4	+
*cox2*	4240	4926	687	229	ATG	TAA	74	+
*trnD*	4951	5015	67				23	+
*atp8*	5084	5245	162	54	ATG	TAG	67	+
*atp6*	5262	5954	693	231	ATG	TAA	16	+
*trnM*	5991	6056	66				36	-
*trnY*	6062	6126	65				5	-
*trnC*	6131	6193	63				4	-
*trnW*	6194	6257	64				0	-
*trnQ*	6261	6313	64				0	-
*trnG*	6325	6389	65				3	-
*trnE*	6390	6455	66				0	-
*rrnS*	6456	7387	932				0	+
*trnV*	7388	7451	65				0	+
*rrnL*	7456	8776	1321				0	+
*trnL1*	8777	8845	69				0	+
*trnL2*	8856	8923	68				10	+
*nad1*	8925	9866	942	314	ATG	TAA	1	+
*trnP*	9867	9930	64				0	+
*nad6*	9932	10436	505	169	ATG	T—	1	+
*cob*	10437	11576	1140	380	ATG	TAG	0	+
*trnS2*	11587	11650	64				10	+
*trnT*	11651	11714	64				0	-
*nad4L*	11722	12018	297	99	ATG	TAG	7	+
*nad4*	12012	13391	1380	460	ATG	TAA	-7	+
*trnH*	13387	13448	62				-5	+
*nad5*	13449	15170	1722	574	ATG	TAA	0	+
*trnF*	15179	15242	64				8	+

^a^ Intergenic nucleotide refer to non-coding bases between two genes, and the negative number indicating gene overlap.

**Table 2:** Genomic characteristics of Conoidea mtDNAs.

Species	Family	Accession number	Whole mitochondrial genome				Protein coding genes			rRNAs		tRNAs		Non-coding regions		Reference
			Length (bp)	A+T%	AT skewness	GC skewness	Length (bp)	A+T%	AT% (3rd)	Length (bp)	A+T%	Length (bp)	A+T%	Length (bp)	A+T%	
Phymorhynchus buccinoides*	Raphitomidae	MN583349	15764	71.14	-0.11	0.03	11262	69.87	84.40	2257	74.57	1446	70.50	519	77.46	this study
*Eubela* sp. ^**●**^	Raphitomidae	MH308406	15153	69.82	-0.11	0.04	11234	74.87	80.97	2255	69.44	1382	66.51	-	-	[2]
*Typhlosyrinx* sp.	Raphitomidae	MH308407	15804	70.43	-0.11	0.04	11255	69.02	81.50	2247	74.19	1440	71.25	-	-	[2]
*Phymorhynchus* sp.	Raphitomidae	MT111940	15631	69.17	-0.12	0.04	11134	67.69	79.72	2256	73.05	1462	69.84	379	74.93	[55]
*Clavatula tripartita*^**●**^	Clavatulidae	MH308391	15743	68.48	-0.11	0.03	11230	70.87	79.03	2327	70.65	1433	68.67	-	-	[2]
*Clionella kraussii*^**●**^	Clavatulidae	MH308390	15760	68.78	-0.11	0.01	11188	71.87	78.90	2341	71.29	1415	68.62	964	72.51	[2]
*Turricula nelliae spurius*	Clavatulidae	MK251986	16453	69.21	-0.10	0.03	11223	68.11	81.72	2235	71.70	1480	69.39	1144	69.14	[56]
*Conus quercinus*	Conidae	MH400188	16380	66.58	-0.15	0.12	11265	72.87	76.08	2328	67.44	1482	64.51	415	62.41	[57]
*Conus capitaneus*	Conidae	NC030354	15829	62.20	-0.18	0.14	11262	73.87	65.50	2206	68.44	1487	65.51	-	-	[54]
*Fusiturris similis*	Fusiturridae	NC_013242	15595	66.37	-0.12	0.04	11244	75.87	73.63	2309	70.44	1485	67.51	-	-	[1]
*Pinguigemmula* sp. ^**●**^	Turridae	MH308408	15250	68.71	-0.11	0.04	11192	68.95	83.00	2319	73.05	1418	69.89	-	-	[2]
*Lophiotoma cerithiformis*	Turridae	DQ284754	15380	67.88	-0.12	0.03	11217	66.68	77.11	2338	71.77	1494	69.41	394	58.88	[58]
*Gemmuloborsonia moosai*	Turridae	MH308392	15541	68.16	-0.12	0.02	11229	67.13	79.27	2318	72.30	1623	68.58	313	68.37	[2]
*Inquisitor* sp. ^**●**^	Pseudomelatomidae	MH308403	15238	67.54	-0.12	0.03	11226	66.53	77.80	2310	70.78	1480	68.24	-	-	[2]
*Otitoma* sp. ^**●**^	Pseudomelatomidae	MH308405	15583	70.10	-0.12	0.04	11229	69.57	84.56	2327	73.49	1493	69.59	920	67.07	[2]
*Oxymeris dimidiata*	Terebridae	NC_013239	16513	65.65	-0.22	0.27	11231	65.02	71.67	2352	65.86	1491	66.53	-	-	[1]
*Splendrillia* sp.1	Drilliidae	MH308395	15358	70.90	-0.11	0.03	11236	70.06	84.90	2342	73.91	1478	69.96	-	-	[2]
*Splendrillia* sp.2 ^**●**^	Drilliidae	MH308396	15231	71.57	-0.11	0.05	11217	70.74	86.70	2344	74.79	1414	70.44	655	72.82	[2]
*Bathytoma punicea*	Borsoniidae	MH308389	16037	65.98	-0.11	0.004	11248	64.85	66.36	2298	70.28	1470	67.96	752	61.57	[30]
*Tomopleura* sp.^**●**^	Borsoniidae	KX263259	15182	69.32	-0.19	0.19	11226	68.11	79.64	2309	73.19	1425	70.46	-	-	[2]
*Anguloclavus* sp. 1 ^**●**^	Horaiclavidae	MH308397	15078	33.78	-0.13	0.10	11216	64.89	68.06	2299	70.81	1403	68.07	-	-	[2]
*Anguloclavus* sp. 2 ^**●**^	Horaiclavidae	MH308399	15103	66.98	-0.05	-0.12	11201	65.77	71.34	2310	71.26	1399	68.33	-	-	[2]
*Benthomangelia* sp. JEU-2016 ^**●**^	Mangeliidae	KX263258	15034	72.4	-0.12	0.02	11221	71.81	72.59	2285	74.31	1384	71.76	-	-	[30]
*Benthomangelia* sp. MNHN IM 2013–9652 ^**●**^	Mangeliidae	MH308400	15037	71.5	-0.12	1.00	11238	70.80	77.20	2285	74.53	1384	71.39	-	-	[2]
*Toxicochlespira* sp. ^**●**^	Mangeliidae	MH308401	15076	72.17	-0.11	0.04	11234	71.71	71.07	2287	74.03	1388	70.61	-	-	[2]
*Cochlespira* sp. ^**●**^	Cochlespiridae	MH308394	15581	63.87	-0.02	-0.17	11200	61.86	66.73	2303	69.65	1416	67.80	-	-	[2]

*Represents the species sequenced by this study

**●** is the mitochondrial genome without complete genes.

### Protein-coding genes

In this study, all the PCGs of *P*. *buccinoides* are located on the positive strand, and this feature is observed in all Conoidea mitochondrial genomes published so far. In the typical metazoan mitochondrial genomes, most PCGs initiate with the standard start codon ATN and terminate with the stop codon TAG or TAA [59]. In *P*. *buccinoides*, all the PCGs are initiated with the ATG codon. For the stop codons, they are ended by a complete TAA (*cox1*, *cox2*, *cox3*, *nad1*, nda2, *nad4*, *nad5*, *nad6*) or TAG (*nad4L*, *atp8*, *cob*, *nad3*), except for *nad6* which is ended with a truncated stop codon T (S4 Table). Similarly, the genes *nad4* and *nad6* in *Eubela* sp. and *Typhlosyrinx* sp. (family Raphitomidae) are also ended with the truncated termination codon T (S4 Table). Previous studies have shown that truncated stop codon is a common phenomenon in the mitochondrial genomes of metazoans [60], and it doesn’t affect the transcription and translation of mitochondrial genes, since the complete stop codon might be obtained by posttranscriptional polyadenylation [16].

**Fig 2:**
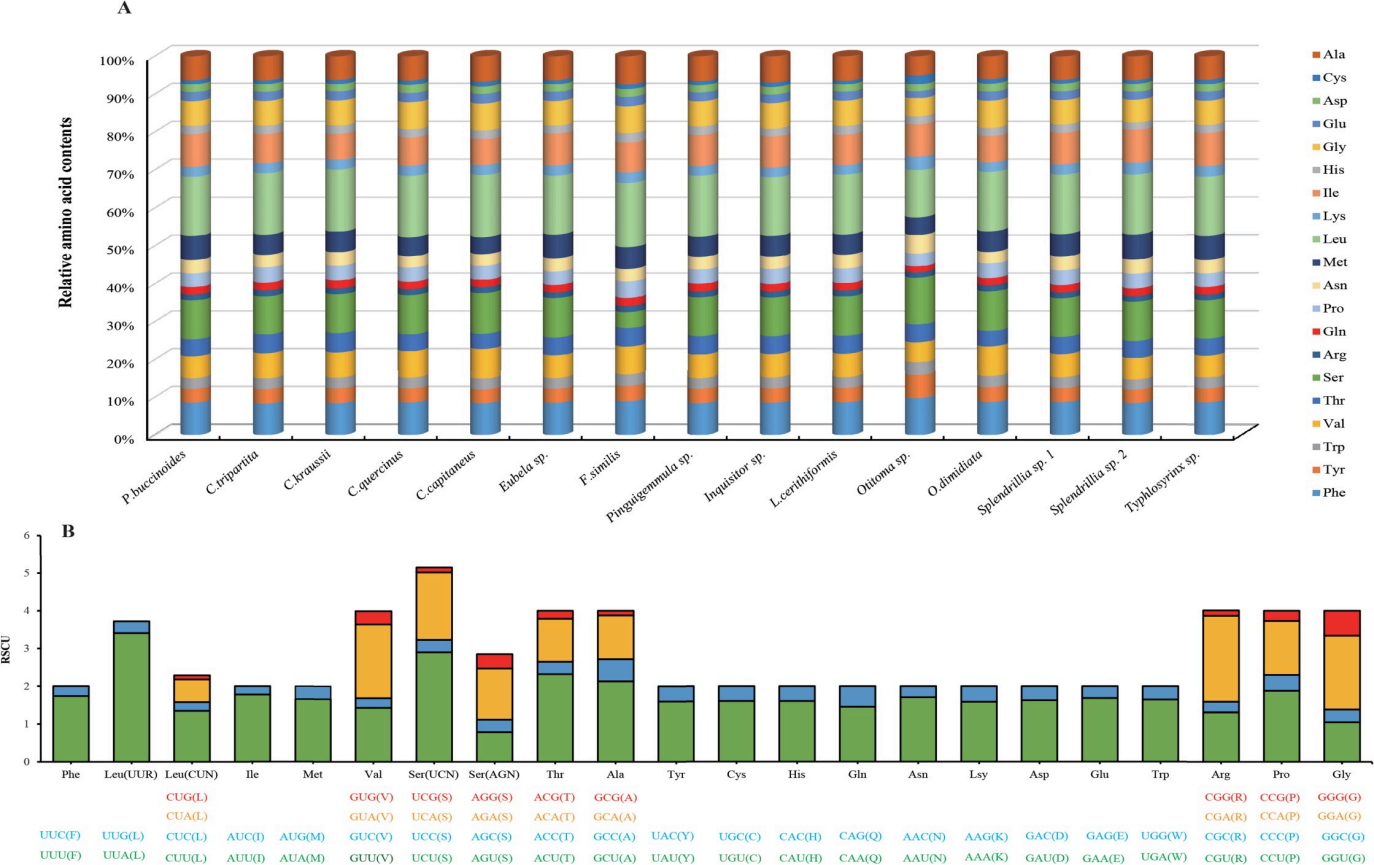
Amino acid contents and codon usage of 13 mitochondrial genes. (A) Relative amino acid contents within the mitochondrial genome of the Conoidea. The X-axis shows the percentage of each amino acid, and the Y-axis shows the name of each species. (B) The relative synonymous codon usage (RSCU) of *P*. *buccinoides* mitochondrial genome. The total number of the RSCU value are provided on the Y-axis, and codon families are on the X-axis.

Previous studies have provided evidence that metazoan mitochondrial genomes usually have different codon usage bias [16, 61]. The amino acid usage and relative synonymous codon usage (RSCU) values in the PCGs of *P*. *buccinoides* are shown in Fig 2. There is a total of 3,741 amino acids (excluding stop codons) in the 13 PCGs of *P*. *buccinoides*, and the amino acid composition is consistent with the other 14 species of Conoidea (Fig 2A). Among PCGs, Leu is the most frequently used amino acid and Cys is the least frequently used, accounting for approximately 15.53% and 1.09% of the total amino acids, respectively. The RSCU indicates the seven most commonly used codons: TTA (Leu), TCT (Ser), GCT (Ala), GTA (Val), CCT (Pro), TCA (Ser), and ATT (Ile) (Fig 2B). Besides, the codons with A and T in the third position are the most frequently used when compared with other synonymous codons. This feature has been observed in many marine invertebrates, such as crab [62], sea cucumber [15], bivalves [16, 63, 64] and gastropods [65, 66].

### Ribosomal RNA and transfer RNA genes

The boundaries of rRNA genes are determined by sequence alignment with that of *Typhlosyrinx* sp. and *Eubela* sp.. As in most Conoidea mitochondrial genomes, the *rrnS* and *rrnL* genes in *P*. *buccinoides* are located between *trnE* and *trnV* and between *trnV* and *trnL*1, respectively (Fig 1).

Based on potential secondary structures, 22 tRNA genes are identified for *P*. *buccinoides*. Generally, a typical cloverleaf of secondary structure in includes an aminoacyl acceptor stem, a TψC stem and loop (T-arm), an anticodon stem and loop, and a DHU stem and loop (D-arm) [11]. Here, all the 22 tRNA genes of *P*. *buccinoides* can be folded into the typical cloverleaf secondary structures (S2 Fig). However, D-stem absence of tRNA genes is common in most Caenogastropoda species [58, 67, 68] and most other metazoans [69–71].

### Gene arrangement

**Fig 3:**
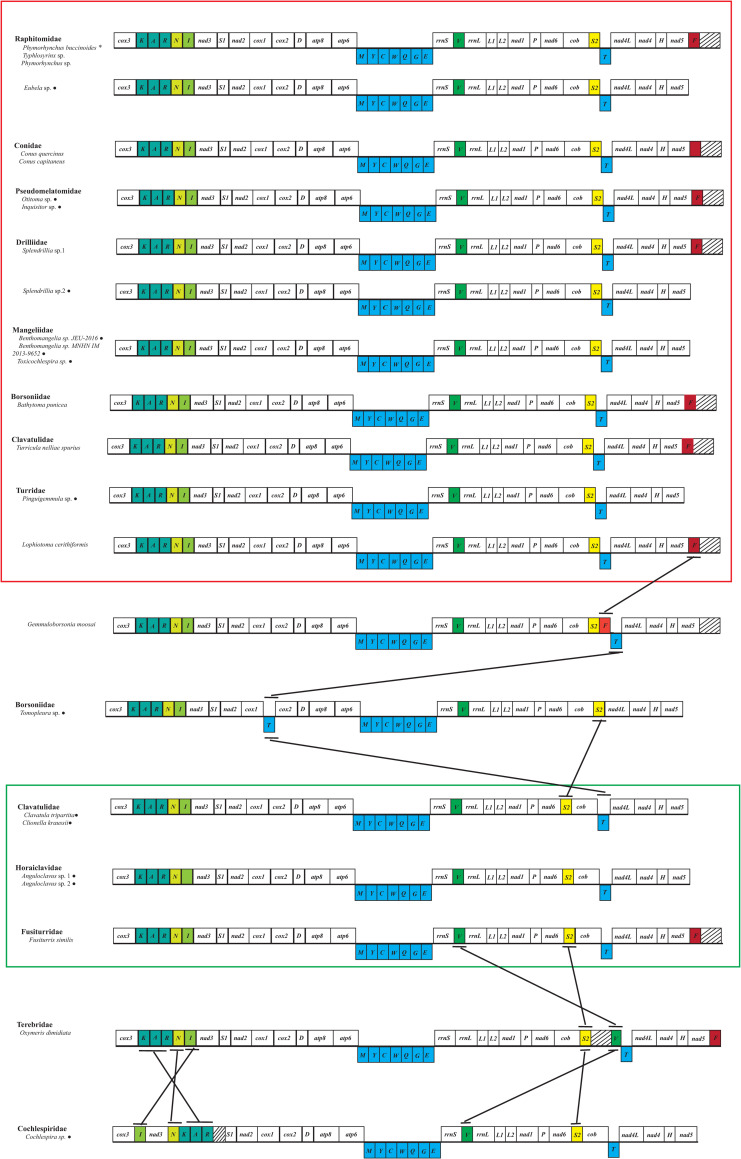
Mitochondrial genome arrangement of 26 species in the superfamily Conoidea. Areas with slashes represent noncoding region. Genes for protein coding genes and rRNAs are shown with standard abbreviation. The tRNA genes are displayed by a single letter for the corresponding amino acid, with two leucine tRNAs and two serine tRNAs differentiated by numerals. The genes above the line are encoded by the heavy strand, while those below the line are encoded by the light strand. Sequence segments are not drawn to scale. *Represents the species sequenced by this study; ^●^ is the mitochondrial genome without complete genes.

Mitochondrial gene arrangements of metazoans are relatively conserved within major lineages but may be variable between them, and comparisons of these gene arrangements have potential for resolving some deep lineage divergences [10, 72]. In the present study, we compared the mitochondrial genome sequence of *P*. *buccinoides* with that of other species in the superfamily Conoidea (Fig 3). All 13 PCGs in Conoidea share the same gene order, while several tRNAs are translocated. The gene order of families Raphitomidae, Conidae, Mangeliidae, Pseudomelatomidae, and Drilliidae (red box in Fig 3) is completely identical. Some species in families Turridae, Clavatulidae and Borsoniidae have the same gene order as Raphitomidae (red box in Fig 3), but tRNA genes in some species of the former three families have been translocated. Comparing the gene order of these eight families (red box in Fig 3) with the species *G*. *moosai* of Turridae shows a translocation of the *trnF* gene from a position between *nad5* and *cox3* to a position between *trnS2* and *trnT*. When these eight families (red box in Fig 3) compared with the species *Tomopleura* sp. of Borsoniidae, the *trnT* gene translocated from a position between *trnS2*and *nad4L* to a position between *cox1* and *cox2*. Comparing the gene order of these eight families (red box in Fig 3) with Terebridae shows a translocation of the *trnV* gene from a position between *rrnS* and *rrnL* to a position between *trnS2* and *trnT*. *TrnK-trnR*, *trnN*, *trnI* and *trnS2* are translocated, when comparing the gene order of these eight families (red box in Fig 3) with Cochlespiridae. The gene order of these eight families (red box in Fig 3) also shows a translocation of the *trnS2* gene from a position between *cob* and *trnT* to a position between *nad6* and *cob* in Clavatulidae, Horaiclavidae and Fusiturridae (green box in Fig 3). There are two tRNA genes translocated, when comparing the gene order of Clavatulidae, Horaiclavidae and Fusiturridae (green box in Fig 3) with Terebridae. One shows a translocation of the *trnV* gene from a position between *rrnS* and *rrnL* to a position between *trnS2* and *trnT*, and the other is the translocation of *trnS2* gene from a position between *nad6* and *cob* to a position between *cob* and *trnV*. There are three and five tRNA genes translocated, when comparing Cochlespiridae with the gene order of Clavatulidae, Horaiclavidae and Fusiturridae (green box in Fig 3) and Terebridae, respectively. These results together with findings from previous studies [1, 73, 74] indicate that the tRNA gene rearrangement of Caenogastropoda occurs occasionally.

### Phylogenetic relationships

**Fig 4:**
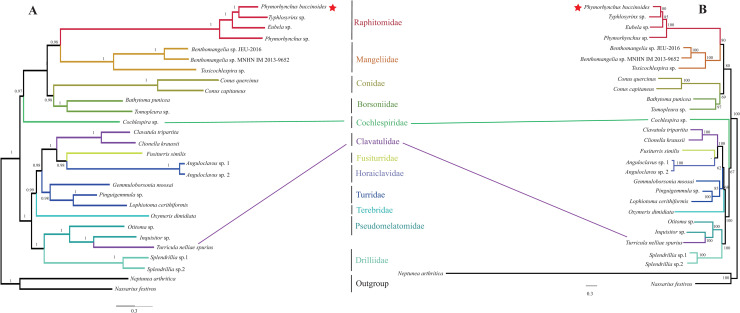
Phylogenetic trees of Conoidea based on nucleotide sequences of 13 concatenated protein-coding genes reconstructed by (A) Bayesian inference and (B) maximum likelihood methods. *Nassarius festivus* and *Neptunea arthritica* are used as outgroup. Numbers at nodes are (A) Bayesian posterior probability and (B) maximum likelihood bootstrap values. The asterisk indicate the species sequenced in this study.

Phylogenetic analysis was performed based on nucleotide sequences of 13 mitochondrial PCGs. The BI and ML analyses generated similar tree topologies with most clades strongly supported (BI posterior probabilities ≥ 0.98; ML bootstrap values ≥ 85%) (Fig 4). The best supported phylogenetic relationship of Conoidea is as follows: (((Raphitomidae + Mangeliidae) + (Conidae + Borsoniidae)) + Cochlespiridae) + ((((Clavatulidae + (Fusiturridae + Horaiclavidae)) + Turridae) + Terebridae) + ((Pseudomelatomidae + Clavatulidae) +Drilidae)). This relationship between Raphitomidae and other Conoidea families is also supported by previous studies [2, 75]. Raphitominae has been recognized as a subfamily of Conidae [76], but a recent study upgrades Raphitominae to a full family [4]. In this study, the result shows that Raphitomidae is separated from Conidae, supporting its distinct role as a family.

### Positive selection analysis

Since the cold seep environments may affect the function of mitochondrial genomes [16], we used positive selection analysis to detect potential selection in cold seep *P*. *buccinoides*. “Branch models” analysis showed no significant (*p* > 0.05) difference between *P*. *buccinoides* and other 22 Conoidea species (ω0 = 0.02215, ω1 = 0.02444) (Table 3). However, when we used the “branch site models” to analyze individual genes, a residue, 18 S in *atp6*, was identified as the positively selected site with high posterior probabilities (BEB values > 95%) (Table 3), suggesting potential positive selection in these amino acid sites.

**Table 3:** Positive selection analysis of the mitochondrial genome of *P*. *buccinoides*.

**Gene**	**Branch models**							**Models compared**		**2ΔlnL**	**LRT p value**
	**Model**	**lnL**	**Estimates of parameters**								
13 PCGs	M1	-121097.61202						M1 VS. M0		205.34830	0.000000
	Two ratio	-121200.28617	ω_0_ = 0.02215 ω_1_ = 0.02444					Two ratio VS. M0		0.48446	0.486409
	M0	-121200.52840	ω = 0.02221								
**Gene**	**Branch-site models**							**Models compared**	**2ΔLnL**	**LRT p value**	**Positive site**
	**Model**	**lnL**	**Estimates of parameters**								
*atp6*	Model A	-7300.15619	site class	0	1	2a	2b	Model A VS. Null model A	2.78491	0.09516	18 S 0.995**
			proportion	0.96472	0.02621	0.00883	0.00024				
			Background ω	0.01539	1.00000	0.01539	1.00000				
			Foreground ω	0.01539	1.00000	5.80659	5.80659				
	Null model A	-7301.548645									

ω = dN/dS; M0: one-ratios model; M1: free-ratios model; Two-ratio: two-ratios model; **Posterior probability > 99%.

The harsh chemosynthetic environment of cold seeps can influence the mitochondrial aerobic respiration [16], and thus survival of cold seep animals may require adaptation of mitochondrial PCGs which play important roles in the oxidative phosphorylation [10, 13]. In the present study, one site of *atp6* is identified to be positively selected. Recent studies have also found positive selection in ATP genes for many deep-sea animals, such as sea anemone [77] and shrimp [25], indicating potential adaptation to marine extreme environments. ATP dehydrogenase not only is the last enzyme complex in the respiratory chain, but also is a part of the regulatory system of complex V [78]. The *atp6* subunit plays an important role in the assembly of F0 proton in ATP synthase, which suggest that mutation in the *atp6* gene may affect the production of ATP [79]. Therefore, we predict that the *atp6* gene may play an important role in *P*. *buccinoides*’s adaptation to cold seep environments.

The deep-sea cold seeps are chemosynthetic ecosystems, which are mainly characterized by high concentrations of methane, sulfide and heavy metals, and low levels of oxygen [80, 81]. These harsh conditions can affect various biological processes, including respiration, reproduction and development [82]. As the energetic centers of eukaryotic cells, mitochondria have proved to be subject to these environmental stress conditions. For example, a previous study based on transcriptomes of shrimps (*Alvinocaris longirostris*) from reducing environments (cold seeps and hydrothermal vents) identified differentially expressed genes including genes associated with mitochondria, which may contribute to adaptation to the harsh conditions [83]. A recent study focusing on vesicomyid clams inhabiting cold seeps and hydrothermal vents found ten potentially adaptive residues in several mitochondrial genes [16]. In this study, one residue in *atp*6, was identified as the positively selected site, suggesting potential adaption of the mitogenome for the cold-seep gastropod *P*. *buccinoides*. Different positively selected genes are detected between this and previous studies [e.g, 16], which may be caused by the fact that different animal groups might have different adaptation mechanisms, or that the results of positive selection are probably inaccurate due to limited species. Nevertheless, more species from cold seeps and other reducing environments such as hydrothermal vents are required to understand the mitochondrial adaptation for this important gastropod group.

## Conclusion

In this study, the complete mitochondrial genome of a cold seep snail, *P*. *buccinoides*, is presented. It is a 15,764 bp circular molecule and encodes 37 typical genes including 13 PCGs, 2 rRNA genes, and 22 tRNA genes. We analyzed the mitochondrial genome content and gene organization, codon usage, gene arrangement, phylogenetic relationships, and positive selection of *P*. *buccinoides*. The mitogenomic features and codon usage of *P*. *buccinoides* are similar to other Conoidea species. We found a completely identical arrangement of PCGs in the mitochondrial genomes of the superfamily Conoidea, when the tRNA genes were not considered. The residue located in *atp6* was inferred to be positively selected. This study is the first determination of the mitochondrial genome of a cold seep member of the Conoidea and may provide evidence for the adaptive evolution of *P*. *buccinoides* in the cold seep environments.

## Supporting information

S1 Fig The morphological image of *P*. *buccinoides*. (EPS)

S2 Fig Secondary structures of tRNAs in the *P*. *buccinoides* mitochondrial genome. The tRNAs are labeled with the abbreviations of their corresponding amino acids.
(EPS)

S1 Table Primers used for amplifying of *P*. *buccinoides* mitochondrial genome. (DOCX)

S2 Table The information of the best fitting substitution model applied to each gene. (DOCX)

S3 Table List of taxa used and the environment of species in the Positive selection analysis. (XLSX)

S4 Table The length, start codon and stop codon of the PCGs of Conoidea. (XLSX)
