# Supplementary material for: The complete mitochondrial genome of a cold seep gastropod Phymorhynchus buccinoides (Neogastropoda: Conoidea: Raphitomidae)
Source: PLoS One. 2020 Nov 30;15(11):e0242541. doi: 10.1371/journal.pone.0242541 (PMC7703994; doi:10.1371/journal.pone.0242541)
Supplement: S1 Table — (DOCX) [file pone.0242541.s003.docx]

**S1 Table. Primers used for amplifying *P. buccinoides* mitogenome.**

| **Short PCR** | | | | | |
| --- | --- | --- | --- | --- | --- |
| **Name** | **Sequence (5’-3’)** | **Region** | **Annealing temperature** | **Product length (bp)** | **Reference** |
| LCO1490 | GGTCAACAAATCATAAAGATATTGG | cox1 | 40℃ | 593 | (20) |
| HC02198 | TAAACTTCAGGGTGACCAAAAAATCA |  |  |  |  |
| 16sinicioF2 | TTCTGCCTGTTTAKCAAAAACATGGCTTC | 16S | 45℃ | 486 | (21) |
| 16sfinR | AAAGATAATGCTGTTATCCCTRCGG |  |  |  |  |
| QW58ND5F4 | TATCACAACAACTTCCCCTAACTAT | nad5 | 50℃ | 728 | In this study |
| QW58ND5R3 | TTAGATGAGGACTATAAAAGCTGAT |  |  |  |  |
| 58tRNAF1 | AGTCTCTCTTCTTCTTTATCTGTCA | nad5 – trnF | 50℃ | 197 | In this study |
| 58tRNAR3 | ATTATACTTTGAGGCAAAGTTGCCA |  |  |  |  |
| 58tRNAF3 | ACTGAAGATGCTGAGGTGGC | trnF – cox3 | 50℃ | 700 | In this study |
| QW58Cox3r10 | GATAACCTTGAAAAGTAGCCTCTCG |  |  |  |  |
| **Long PCR** | | | | | |
| **Name** | **Sequence (5’-3’)** | **Region** | **Annealing temperature** | **Product length (bp)** | **Reference** |
| Cdeacox3F | ATGGCACGAAATCCATTTCATTTRGTTGA | cox3 – cox1 | 45℃ | 3081 | (2) |
| Raphi COIR | ACAGCYCCTAAAATWGAAGAWACACCAGC |  |  |  |  |
| Raphi COIF | GGTGCTCCAGATATRGTWTTTCCTCG | cox1 – 16S | 45℃ | 5351 | (2) |
| Cdea16sR | CTACCTTTGCACGGTCAGAGTACC |  |  |  |  |
| Cdea16sF | GCCTTATAATTGAAGGCTRGWATGAATGG | 16S – trnF | 45℃ | 6226 | (2) |
| LuoLND5R1 | TAGTTAGGGGAAGTTGTTGTGATA |  |  |  | In this study |
