# Supplementary material for: The complete mitochondrial genome of a cold seep gastropod Phymorhynchus buccinoides (Neogastropoda: Conoidea: Raphitomidae)
Source: PLoS One. 2020 Nov 30;15(11):e0242541. doi: 10.1371/journal.pone.0242541 (PMC7703994; doi:10.1371/journal.pone.0242541)
Supplement: S2 Table — (DOCX) [file pone.0242541.s004.docx]

**S2 Table. The best substitution model applied to each gene.**

| **Gene** | **Model** |
| --- | --- |
| atp6 | TVM+I+G |
| atp8 | HKY+I+G |
| cox1 | GTR+I+G |
| cox2 | TPM3uf+I+G |
| cox3 | TVM+I+G |
| cob | TVM+I+G |
| nad1 | TVM+I+G |
| nad2 | TVM+G |
| nad3 | TIM3+I+G |
| nad4 | TVM+I+G |
| nad4L | TIM2+I+G |
| nad5 | TVM+I+G |
| nad6 | TVM+I+G |
